# SRM Represents a Novel Prognosis Biomarker and Correlates With Inflammation and Immune Infiltration in Hepatocellular Carcinoma

**DOI:** 10.1155/mi/6025289

**Published:** 2026-07-13

**Authors:** Bo-wen Wu, Feng-hong Wang, Lei Zhang, Ting Li, Jian-qiang Zhang, Sheng-bo Yang, Xue-mei Wang, Ya-nan Li

**Affiliations:** ^1^ Department of Health Statistics, School of Public Health, Inner Mongolia Medical University, Hohhot, China, immu.edu.cn; ^2^ Department of Toxicology, School of Public Health, Inner Mongolia Medical University, Hohhot, China, immu.edu.cn; ^3^ Department of Occupational Health and Environmental Health, School of Public Health, Binzhou Medical University, Yantai, China, bzmc.edu.cn; ^4^ Department of Occupational Health and Environmental Health, School of Public Health, Inner Mongolia Medical University, Hohhot, China, immu.edu.cn

**Keywords:** bioinformatics, hepatocellular carcinoma, immune infiltrate, inflammation, SRM

## Abstract

Hepatocellular carcinoma (HCC) is widely recognized as one of the leading causes of cancer‐related deaths worldwide. Although advances in screening, diagnosis, and treatment have been made, reliable biomarkers are urgently needed to monitor the disease. This study aims to investigate the association between spermidine synthase (SRM) and clinicopathological characteristics, inflammatory responses, and immune infiltration in HCC. RNA‐seq data and clinical information for liver HCC (LIHC) were obtained from the Gene Expression Omnibus (GEO) and The Cancer Genome Atlas (TCGA) databases to assess SRM expression. The correlation between SRM expression and immune infiltration was analyzed using the TIMER algorithm. Comprehensive analyses of immune checkpoints (ICPs), microsatellite instability (MSI), and tumor mutational burden (TMB) were performed using R‐based packages. KEGG analysis indicated SRM is involved in the IL‐17 signaling pathway. This association was further supported by experimental validation of key markers via qPCR and western blot. Functional studies, including in vivo experiments, are needed to establish a causal relationship. Our results show that SRM expression is significantly elevated in HCC tissues compared to adjacent nontumor tissues and associated with adverse clinicopathological features and poor prognosis. SRM expression was significantly correlated with immune infiltration levels in LIHC, involving 22 immune cell subtypes, particularly tumor‐associated macrophages (TAMs; CD86 and IL10). Moreover, SRM expression was closely associated with ICPs, TMB, and MSI. Based on transcriptomic data, KEGG pathway analysis of SRM‐associated differentially expressed genes revealed significant enrichment in the IL‐17 signaling pathway. These in vitro findings suggest a potential association among SRM, IL‐8 expression, and pathways related to tumor progression and immune modulation, although further in vivo studies are required to confirm these observations. However, in vivo studies using animal models are required to evaluate whether targeting SRM has therapeutic effects on HCC and to further validate these mechanistic findings.

## 1. Introduction

Hepatocellular carcinoma (HCC) makes up approximately 90% of primary liver cancer cases and is one of the three leading causes of cancer‐related deaths worldwide [1]. The pathogenesis of HCC involves multiple factors, including complex interactions among viral/nonviral etiologies, genetic susceptibility, immune dysregulation, and alterations in the tumor microenvironment (TME) [2]. Current treatment strategies for HCC primarily include surgical interventions, locoregional treatments, and systemic therapies. Although significant advances have been made in diagnostic methods, the prognosis for patients with HCC remains poor [3]. Notably, breakthroughs in immunology research have revolutionized cancer treatment, enabling a significant proportion of patients across various cancer types to achieve durable clinical responses. Among these breakthroughs, immune checkpoint (ICP) inhibitors against cytotoxic T lymphocyte‐associated protein 4 (CTLA‐4) and programmed cell death protein 1 (PD‐1) have become key immunotherapies [4]. However, with the widespread clinical use of ICP inhibitors, the search for reliable biomarkers to predict treatment efficacy remains urgent.

Spermidine synthase (SRM) is a key enzyme involved in eukaryotic cell growth, which is closely associated with tumor progression, invasion, and metastasis [5]. Polyamine metabolic dysregulation is a hallmark of cancer cells [6]. As a key enzyme in polyamine biosynthesis, SRM catalyzes the production of spermidine, a polyamine ubiquitous in all living organisms that is essential for cellular function [7]. Notably, spermidine plays a critical role in the activation of eIF5A, which has previously been considered a potential therapeutic target for malarial treatment [8]. Recent evidence indicates that the lipolytic factor ABHD5 promotes colorectal cancer (CRC) progression by regulating SRM‐dependent spermidine synthesis in tumor‐associated macrophages (TAMs), thereby establishing the ABHD5/SRM/spermidine axis as a highly promising therapeutic target in TAMs [9]. Furthermore, studies have shown that inhibiting SRM reduces spermidine levels in OVCAR3 cells, thereby confirming the association between SRM and immune infiltration in cyclin E1‐driven ovarian cancer [10]. Hypoxia triggers the specific enrichment of SRM in HCC‐derived exosomes. Upon uptake of SRM from these exosomes by TAMs, SRM promotes their polarization toward M2‐type macrophages via the eIF5A‐hypusination/oxidative phosphorylation (OXPHOS) axis, thereby creating an immunosuppressive microenvironment [11]. However, the exact relationship between SRM and immune infiltration/inflammation in HCC remains unclear. To address this knowledge gap, we employed a comprehensive multiomics approach to evaluate the association between SRM and key clinical characteristics, including disease stage, prognosis, and response to immunotherapy.

To this end, we first assessed the relevance of SRM to key clinical features, including disease stage, prognosis, and immunotherapy response. We further explored the interplay between SRM expression and the IL‐17 pathway, while elucidating its relationship with immune cell infiltration and inflammatory responses. Ultimately, our study aimed to investigate whether and how SRM regulates the IL‐17 signaling pathway to promote HCC progression and immunotherapy resistance. These findings may provide valuable insights.

## 2. Methods

### 2.1. Data Collection

The Cancer Genome Atlas (TCGA) GDC database (https://portal.gdc.cancer.gov/) was accessed to acquire the SRM expression and corresponding clinical information. Two modules—“cases” and “files”—were used for data search and selection. First, the following keywords were applied in the case module: “liver and intrahepatic bile ducts [Primary Site],” “TCGA [Program],” “TCGA‐LIHC [Project],” and “Adenomas and Adeno carcinomas [Disease Type].” Second, the following keywords were applied in the files module: “Transcriptome Profiling” for [Data Category], “Gene Expression Quantification” for [data type], “STAR‐Counts” for [Workflow Type], and “RNA‐Seq” for [experimental strategy]. RNA‐seq count matrices were processed using Strawberry Perl software (version 5.32.1.1, https://strawberryperl.com/).

Gene expression and corresponding clinical data of HCC patients were downloaded from the Gene Expression Omnibus (GEO) database (https://www.ncbi.nlm.nih.gov/geo/). The search keywords were “liver cancer” and “survival,” with the following filters: “Series” for entry type, “Expression profiling by array” for study type, and “*Homo sapiens*” for organism. We selected the gene expression microarray datasets GSE54236 and GSE112790, as these two datasets contain the largest number of HCC samples. Using the GPL10558 platform, probe IDs were matched to gene symbols. For genes with multiple corresponding probes, the probe‐level expression values were averaged to obtain a single gene‐level expression value.

### 2.2. SRM Gene and Survival Analysis

To explore the prognostic relevance of SRM in HCC specimens, we retrieved the corresponding clinical datasets from the TCGA–liver HCC (LIHC) database. Kaplan–Meier curves were plotted using the survival and survminer R packages, and time‐dependent receiver operating characteristic (ROC) analysis was performed using the timeROC R package to evaluate the accuracy of the SRM‐based risk score in predicting prognosis [12, 13] (Supporting Information 1: Table S1).

### 2.3. SRM With Coexpression Genes and Functional Enrichment Analysis

We screened for genes coexpressed with SRM by cor. test using the Spearman method. Coexpression analysis was used in the ggplot, ggpubr, and ggExtra packages. The coexpression circle diagram was generated using the corrplot and circlize packages to enrich genes interacting with SRM [14] (Supporting Information 2: Table S2). Genes with high positive or negative correlation coefficients with SRM were first filtered and then analyzed using clusterprofiler package with the enrichGO, gene set enrichment analysis (GSEA), and enrichKEGG function for KEGG and Gene Ontology (GO) enrichment analysis, respectively. These analyses were visualized using the colorspace, stringi, and ggplot2 packages [15].

### 2.4. SRM and TME of HCC

To investigate the TME of SRM in HCC samples, we accessed the “estimate” package to estimate the SRM of the immune score and stromal cell score [16] (Supporting Information 3: Table S3).

### 2.5. Correlation Analysis of SRM Expression With Immune Infiltration, Tumor Mutational Burden (TMB), and Microsatellite Instability (MSI) Results

First, we obtained the immuno‐infiltration results file via the installation of the “preprocess Core” package (Supporting Information 4: Table S4). To conduct a reliable immune assessment [17], we estimated the immune scores using the infiltration landscape using the CIBERSORT algorithm and visualized the results using the “ggplot2” and “heatmap” packages in R. We set *p* < 0.05 as the screening criterion and identified 22 types of immune cells that might be influenced by SRM expression. Second, we analyzed the correlation between SRM expression and the infiltration levels of these immune cells in HCC samples [18]. The TIMER and gene expression profiling interactive analysis (GEPIA) online tools were used to analyze the correlation between SRM expression and tumor‐associated macrophage markers (CD86 and IL10), respectively. Correlation analysis of SRM and immune cells was performed by coexpression analysis (Supporting Information 5: Table S5). In addition, we analyzed genes associated with ICPs through the Wilcoxon test (Supporting Information 6: Table S6) [19]. Spearman’s correlation analysis revealed significant associations between SRM and TMB [20] (Supporting Information 7: Table S7), as well as between SRM and MSI [21] (Supporting Information 8: Table S8). A *p*‐value of 0.05 was considered statistically significant.

### 2.6. Cell Lines, Cell Culture, and Transfection

HepG2, PLC/PRF/5, Hep3B, and human liver normal cell line L‐02 were obtained from the China Cell Collection (Shanghai, China). Cells were cultured in the incubator at 37°C and 5% CO_2_ in DMEM medium supplemented with 10% fetal bovine serum. HepG2 cells were treated with IL‐17 (Beyotime, P5205) to activate the SRM expression. SRM‐targeting small interfering RNA (siRNA) was synthesized by GenePharma (China). For transfection, human siSRM was co‐introduced into the cultured cells using Lipofectamine 2000 (Invitrogen) and a matching volume of siRNA suspension, following standard laboratory protocols (siRNA sequences are detailed in Supporting Information 9: Table S9).

### 2.7. Western Blot Analysis

Western blot analysis was carried out as previously described. The primary antibodies used were anti‐SRM (19858‐1‐AP, propteintech, 1:1000), anti‐IL‐17 (13082‐1‐AP, propteintech, 1:1000), anti‐IL‐17A (26163‐1‐AP, propteintech, 1:1000), anti‐ACT1 (26692‐1‐AP, propteintech, 1:1000), anti‐TRAF6 (83535‐2‐RR, propteintech, 1:1000), anti‐IL‐8(27095‐1‐AP, propteintech, 1:1000), anti‐p38(14064‐1‐AP, propteintech, 1:1000), anti‐p‐p38(28796‐1‐AP, propteintech, 1:1000), anti‐p65(66535‐1‐Ig, propteintech, 1:1000), anti‐NF‐κB(8242 T, CST, 1:1000), anti‐β‐actin (81115‐1‐RR, propteintech, 1:10,000). Goat anti‐mouse IgG (H + L), HRP conjugate (ZB‐2305, ZSGB‐BIO, 1:10,000) and Goat anti‐rabbit IgG (H + L), and HRP conjugate (ZB‐2301, ZSGB‐BIO, 1:10,000) were used as secondary antibodies.

### 2.8. RNA Extraction and Quantitative PCR Assay

The manufacturer’s instructions were followed to isolate total RNA using Trizol (Sigma–Aldrich, T9424), reverse‐transcribe it using the Fastking RT Kit (TIANGEN Biotechnology [Beijing], KR116−02), and an oligo‐dT primer. Quantitative PCR experiments were performed using the SYBR Green PCR Master Mix and an ABI 7500 Fast real‐time PCR system (Applied Biosystems), in accordance with the manufacturer’s instructions. All primers in real‐time qPCR are listed in Supporting Information 10: Table S10.

### 2.9. Co‐Immunoprecipitation (Co‐IP) Assays

Co‐IP assays were routinely performed as previously described [22].

### 2.10. Immunofluorescence Assay

The immunofluorescence assay was routinely performed as previously described [22].

### 2.11. Statistical Analysis

The Wilcoxon signed‐rank or Mann–Whitney *U* tests were used to compare gene expression profiles between two groups of HCC samples. Survival, functional enrichment, and TMB were performed using R version 4.2.0. Scatter plots were generated using the ggpubr R package to assess the correlation between the SRM mRNA expression and immune‐related genes. Statistical analysis was performed using GraphPad Prism 9.1. All in vitro experiments were repeated three times, and data are presented expressed as the mean ± SD. Comparisons between two groups in vitro were analyzed using the student’s *t*‐test. A *p*‐value < 0.05 was considered statistically significant.

## 3. Results

### 3.1. Expression Profiles of SRM in HCC Patients

**Figure 1:**
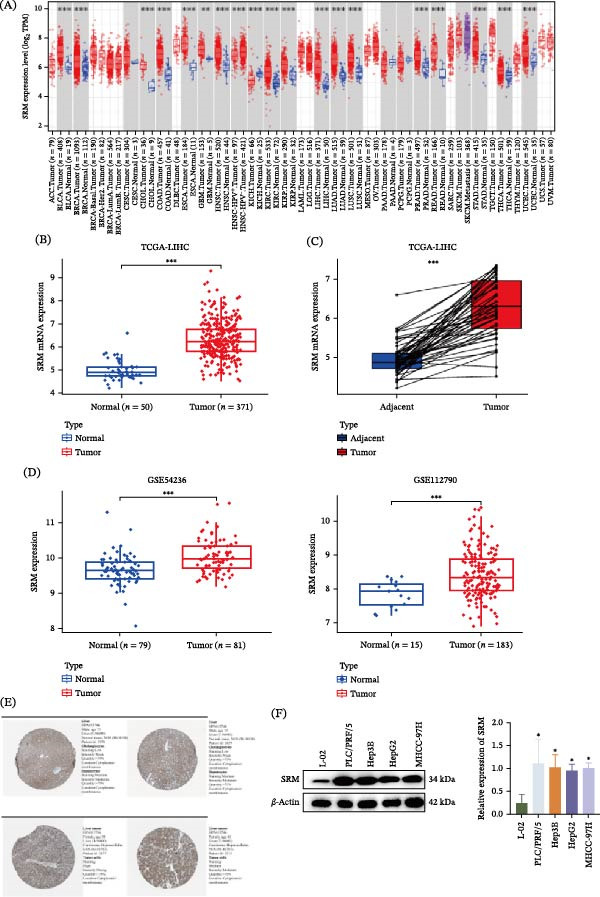
Expression profiles of SRM in HCC tissues. (A) SRM expression profiles across various pan‐cancers were analyzed using the TIMERdatabase.  ^∗∗^ *p*< 0.01,  ^∗∗∗^ *p*< 0.001 vs. normal group. (B) Comparative analysis of SRM mRNA levels between HCC and normal liver tissues from TCGA‐LIHC dataset.  ^∗∗∗^ *p*< 0.001 vs. normal group. (C) SRM mRNA expression levels in paired samples of adjacent normal tissues (*n* = 50) and tumor tissues (*n* = 50) from TCGA‐LIHC dataset.  ^∗∗∗^ *p*< 0.001 vs. normal group. (D) Validation of SRM mRNA expression patterns in HCC versus normal liver tissuesusing GSE54236 and GSE112790 datasets.  ^∗∗∗^ *p*< 0.001 vs. normal group. (E) Representative immunohistochemistry images of SRM expression in malignant and normal liver tissues, sourced from The Human Protein Atlas database. (F) Differential SRM expression between L02 and HCC cell lines.  ^∗^ *p*< 0.05 vs.L02 group.

We first analyzed TCGA RNA‐seq data using the TIMER 2.0 database and determined the mRNA levels of SRM transcripts across different cancer types. SRM exhibited significantly higher expression in the liver cancer tissues than in normal liver tissues. SRM was also highly expressed in the cancer tissues, including breast invasive carcinoma (BRCA), bladder urothelial carcinoma (BLCA), and cholangiocarcinoma (CHOL). However, kidney chromophobe (KICH) cancer tissues showed a low level of SRM (Figure 1A). In the TCGA–LIHC dataset, SRM expression was significantly higher in the liver malignant tissues than in the normal liver tissues (Figure 1B) and in the 50 paired tumor and adjacent samples from the TCGA–LIHC dataset (Figure 1C). GEO data also identified higher SRM mRNA levels in liver cancer tissues as compared with those in normal liver tissues (Figure 1D). The earlier SRM expression was confirmed by analyzing the Human Protein Atlas (HPA) data in liver patients and in HCC cells, as indicated in Figure 1E,F.

### 3.2. The Relationships Between SRM and Clinicopathological Characteristics of HCC Patients

**Figure 2:**
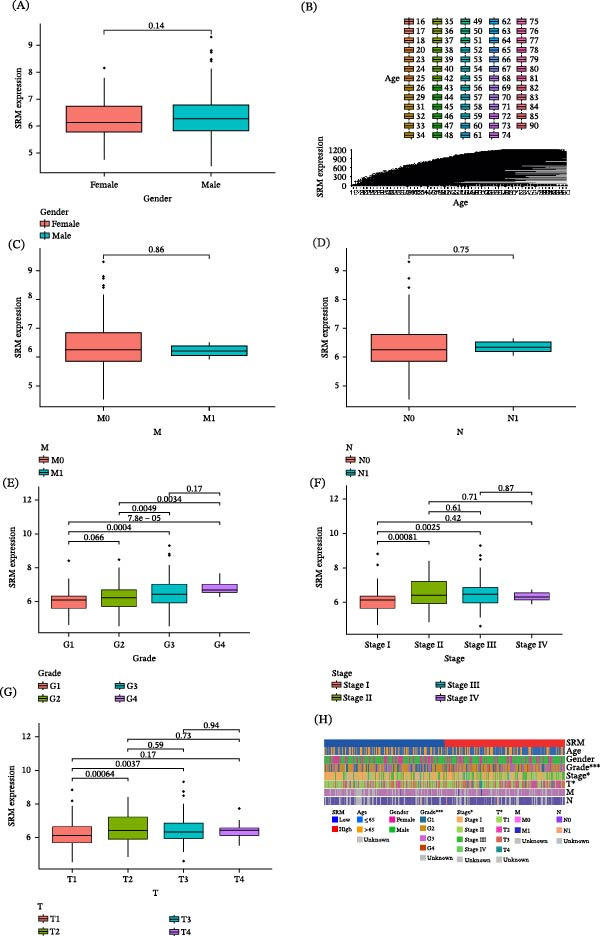
SRM expression in relation to clinicopathological features of HCC patients. (A–G) SRM expression levels stratified by (A) gender, (B) age, (C) distant metastasis, (D) lymph node metastasis, (E) tumor grade, (F) disease stage, and (G) invasion depth. (H) Correlation analysis revealed that SRM expression was significantly associated with tumor stage, grade, and invasion depth in HCC.  ^∗^ *p*< 0.05,  ^∗∗∗^ *p*< 0.001 vs. SRM‐low group.

SRM and clinical correlation characteristics, including age, gender, stage, grade (G), lymph node metastasis (N), invasion depth (T), and distant metastasis (M), were analyzed in HCC patients by using the TCGA‐LIHC dataset with univariate Cox regression analysis. The clinical characteristics of HCC patients with low and high expression of SRM from the TCGA–LIHC dataset are shown in Table 1. As shown in Figure 2A–D, there was no significant relevance between SRM expression and other clinicopathological characteristics, including gender (*p* = 0.14), age, distant metastasis (*p* = 0.86), and lymph node metastasis (*p* = 0.75). However, high expression of SRM was significantly associated with stage and grade (*p* < 0.05) (Figure 2E–G). These findings demonstrated a relationship between SRM expression and stage, grade, and depth of invasion of HCC patients (Figure 2H).

**Table 1:** The clinical characteristics of HCC patients with low and high expression of SRM from the TCGA‐LIHC dataset.

Clinical characteristic (371)	High expression (*N* = 176)	Low expression (*N* = 195)	*p*‐Value
Gender, *n* (%)			
Female	47 (26.7%)	74 (37.9%)	0.028093
Male	129 (73.3%)	121 (62.1%)	
Age, *n* (%)			
<60	84 (47.7%)	85 (43.6%)	0.48726
≥60	92 (52.3%)	110 (56.4%)	
Race, *n* (%)			
Asian	2 (1.1%)	0 (0%)	0.14995
Black	79 (44.9%)	79 (40.5%)	
African	11 (6.3%)	6 (3.1%)	
American	3 (1.7%)	7 (3.6%)	
White	81 (46.0%)	103 (52.8%)	
N stage, *n* (%)			
N0	123 (69.9%)	129 (66.2%)	0.56161
N1	1 (0.6%)	3 (1.5%)	
N2	52 (29.5%)	62 (31.8%)	
N3	0 (0%)	1 (0.5%)	
M stage, *n* (%)			
M0	130 (73.9%)	136 (69.7%)	0.65778
M1	2 (1.1%)	2 (1.0%)	
MX	44 (25.0%)	57 (29.2%)	
T stage, *n* (%)			
T1	71 (40.3%)	110 (56.4%)	0.023889
T2	52 (29.5%)	44(22%)	
T3	30 (17.0%)	15 (7.7%)	
T3a	14 (8.0%)	15 (7.7%)	
T3b	3 (1.7%)	3 (1.5%)	
T4	6 (3.4%)	7 (3.6%)	
Pathologic_stage, *n* (%)			
Stage I	76 (42.3%)	119 (61%)	0.023393
Stage II	49 (27.8%)	37 (19.0%)	
Stage IIIA	40 (22.7%)	25 (12.8%)	
Stage IIIB	5 (2.8%)	3 (1.5%)	
Stage IIIC	3 (1.7%)	6 (3.1%)	
Stage IV	1 (0.6%)	1 (0.5%)	
Stage IVA	1 (0.6%)	0 (0%)	
Stage IVB	1 (0.6%)	1 (0.5%)	
Stage III	0 (0%)	3 (1.5%)	

### 3.3. Prognostic Value of SRM in HCC Patients

**Figure 3:**
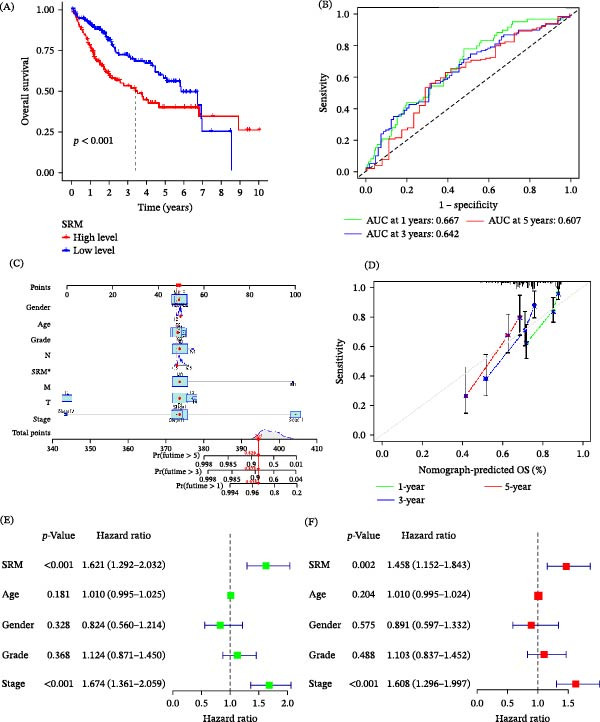
Prognostic value of SRM expression in HCC patients. (A) Kaplan–Meier curve showing OS of HCC patients stratified by high vs. low SRM expression. (B–D) Time‐dependent ROC curves evaluating the predictive performance of SRM expression for 1‐, 3‐, and 5‐year OS. SRM ^∗^ refers to the cox regression‐calibrated stratified variable of SRM gene expression. (E) Univariate Cox regression analysis for OS using various clinicopathological variables. (F) Multivariate Cox analysis for OS, adjusted for significant variables from univariate analysis.

We analyzed the predictive value of SRM in HCC patients. The expression level of SRM showed worse overall survival (OS) in the SRM‐high group than in the SRM‐low group from the TCGA–LIHC dataset (Figure 3A). In addition, we used the ROC curve to predict HCC patients’ survival. The ROC curve analysis showed that 1‐, 3‐, and 5‐year OS rates were 0.667, 0.642, and 0.607, respectively (Figure 3B). As shown in Figure 3C,D, the line graph demonstrated that high SRM expression was associated with poorer OS in HCC patients. Next, univariate Cox regression analyses demonstrated a relationship between OS and illness stages as well as SRM expression (*p* < 0.001, HR = 1.621, 95% CI = 1.292–2.032). (Figure 3E). Additionally, multivariate Cox regression demonstrated that SRM expression was an independent prognostic predictor (*p* < 0.01, HR = 1.458, 95% CI = 1.152–1.843) (Figure 3F). Overall, our findings indicated that SRM was a risk factor for poor prognosis and a standalone prognostic marker.

### 3.4. SRM Gene Significantly Correlated With HCC Tumor Infiltration

**Figure 4:**
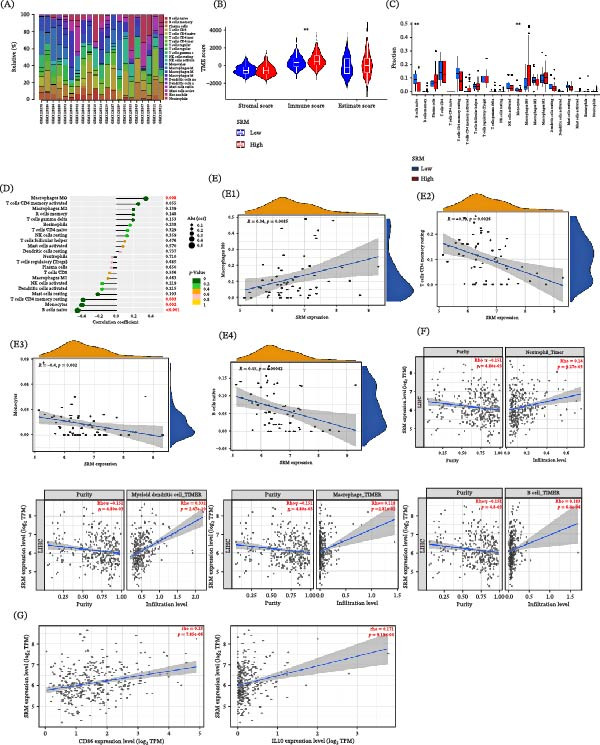
Analysis of SRM expression with the immune filtration in HCC. (A) Percentage abundance of tumor‐infiltrating immune cells in HCC samples. (B) Compare the immune score, stromal scores, and ESTIATE score between the SRM‐high and SRM‐low groups using the ESTIMATE algorithm.  ^∗∗^ *p*< 0.01 vs. SRM‐low group. (C) Compare immune cell filtration between 22 immune cells and SRM expression in the SRM‐high and SRM‐low groups.  ^∗∗^ *p*< 0.01 vs. SRM‐low group. (D) Correlation matrix of the difference between immune cells and SRM in HCC. (E) Scatterplots of the correlations between SRM expression and immune cells of (E1) M0 macrophages, (E2) T cells, CD4 memory resting, (E3) monocytes and, (E4) B cells native were shown. (F, G) Scatterplots showing purity‐corrected correlations of SRM expression with tumor‐infiltrating immune cell levels and TAM markers (CD86 and IL10).

Given that immune cell infiltration has been confirmed to be closely associated with tumor progression and therapeutic efficacy, we speculate that SRM may influence patient prognosis by modulating the tumor immune microenvironment [23, 24]. First, we analyzed the relative expression levels of SRM in different types of HCC‐infiltrated immune cells (Figure 4A). To determine the relationship between SRM and TME, we used the CIBERSORT algorithm to estimate the immune cell infiltration landscape. The ESTIMATE algorithm was subsequently applied to calculate the immune score and stromal score between the SRM‐high and SRM‐low groups. As shown in Figure 4B, the immune score of the SRM‐high group was significantly higher than that of the SRM‐low group. We then compared the relative abundance of 22 types of tumor‐infiltrating immune cells between the SRM‐high and SRM‐low groups. The abundance of B cells naive and monocytes exhibited lower proportions in the SRM‐high group as compared to the SRM‐low group (*p* < 0.01). Conversely, the proportion of macrophage M0 cells was upregulated in the SRM‐high group (Figure 4C). From Figure 4D,E, the SRM had a positive correlation with macrophages M0 (*R* = 0.34, *p* < 0.01), but with a negative correlation with T cells CD4 memory resting (*R* = −0.39, *p* < 0.01), and monocytes (*R* = −0.4, *p* < 0.01). The “gene” panel in TIMER was used to investigate the relationship between SRM and immune‐ infiltrating levels. Results indicated that SRM was positively correlated with B cells (*R* = 0.183, *p* < 0.001, myeloid dendritic cell (*R* = 0.332, *p* < 0.001), macrophage (*R* = 0.118, *p* < 0.05), neutrophil (*R* = 0.14, *p* < 0.01), but negatively correlated with tumor purity in HCC (*R* = −0.151, *p* < 0.01) (Figure 4F). After adjusting for tumor purity, the results showed that TAM markers such as CD86 and IL10 exhibited moderate correlations with the SRM expression (Figure 4G). Overall, SRM expression showed differential correlations with various tumor‐infiltrating immune cell subsets in HCC, with positive correlations observed for macrophages M0 and neutrophils and negative correlations for B cells and monocytes.

### 3.5. SRM Expression Was Related to the ICP Genes, TMB, and MSI in HCC

**Figure 5:**
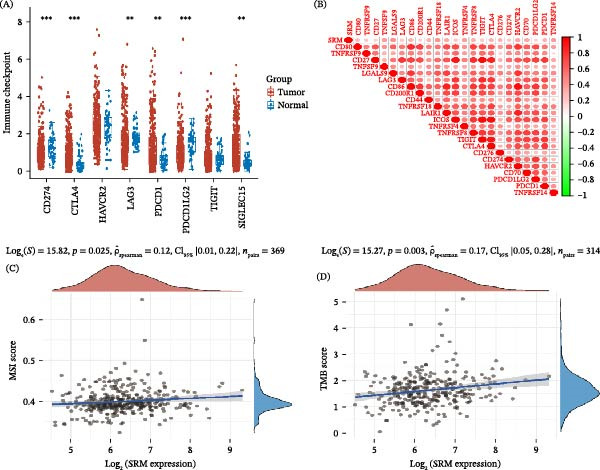
Immune checkpoint expression profile and its correlation with SRM in HCC. (A) Distribution of immune checkpoint gene expression in HCC and normal tissues.  ^∗∗^ *p* < 0.01,  ^∗∗∗^ *p*< 0.001 vs. normal tissues. (B) Heatmap of SRM and immune checkpoint‐associated gene expression. (C) Correlation between SRM and MSI in HCC. (D) Correlation between SRM and TMB in HCC.

The expression of the SRM gene was significantly correlated with tumor infiltration in HCC. Given this, we further investigated the potential functions of SRM within the TME. Existing studies indicate that the ICP genes play a critical role in modulating immune cell infiltration as well as the outcomes of immunotherapy [25]. First, the distribution of ICP genes showed that the expression levels of CD274, CTLA‐4, LAG‐3, PDCD1, PDCD1LG2, and SIGLEC‐15 were higher in HCC tissues compared with those in normal liver tissues based on the TCGA–LIHC dataset (Figure 5A). We then explored the association between SRM and ICP genes in human cancers to discover the potential of SRM in immunotherapy. As shown in Figure 5B, 23 ICP genes were related to SRM expression in different signal transduction pathways. This suggested that SRM expression is closely associated with the ICP gene signature, implying its potential relevance as a biomarker for immunotherapy. We hypothesized that SRM could predict immunotherapy response or produce a favorable therapeutic outcome when used as a possible HCC biomarker or as a new immunotherapy target. TMB serves as a biomarker to predict the efficacy of immunotherapy in CRC [26]. MSI is considered a predictive biomarker for cancer immunotherapy [27]. To explore the prognostic value of SRM in relation to TMB and MSI, we observed a correlation between SRM and TMB (*p* < 0.05) (Figure 5D) and between SRM and MSI (*p* < 0.05) (Figure 5C). The expression of SRM showed significant correlations with ICP genes, TMB, and MSI, suggesting its potential role in regulating the immune response in HCC.

### 3.6. Analysis of Genes Coexpressed and the Pathways With SRM in HCC

**Figure 6:**
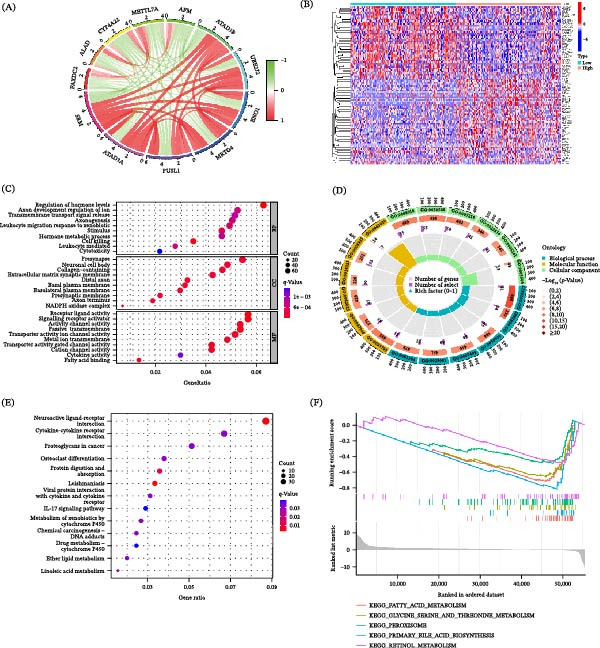
SRM coexpressed genes and functional enrichment analysis. (A) Circle plot showing the coexpression profile genes with SRM gene in HCC. The top five positively and five negatively correlated genes are displayed. (B) Heatmap of differential expressed genes with SRM. The top 30 positively and 30 negatively correlated genes are displayed. (C, D) GO enrichment analysis of SRM‐associated differentially expressed genes. The top 10 terms for each GO subtype are shown. (E) KEGG pathway enrichment analysis of SRM‐associated differentially expressed genes. The top 10 enriched pathways are shown. (F) GSEA revealing key pathways associated with SRM expression.

To further investigate the broader functional networks involving SRM, we analyzed genes coexpressed with SRM and the pathways enriched in HCC. Coexpressed genes with SRM were identified utilizing a corrplot, which served as the basis for subsequent pathway enrichment analysis to explore the potential functional influence of SRM in HCC. SRM showed a top positive correlation with ATAD3A, ATAD3B, PUSL1, MRTO4, ENO1, and other molecules (Figure 6A, Supporting Information 11: Figure S1). We screened the differential genes with SRM in the SRM‐high and SRM‐low groups in the TCGA–LIHC dataset for functional enrichment analysis (Figure 6B). GO analysis showed that differentially expressed genes were enriched in biological processes such as regulation of hormone levels (*p* < 0.001), and signaling receptor activator (*p* < 0.001), as well as presynapse (*p* < 0.001) (Figure 6C,D). GSEA showed that the retinol metabolism enrichment score was enriched in the SRM‐high group. In contrast, pathways involved in fatty acid metabolism, threonine metabolism, glycine‐serine metabolism, peroxisome function, and primary bile acid biosynthesis were predominantly enriched in the SRM‐low group (Figure 6F). Meanwhile, KEGG enrichment analysis showed that these genes were closely associated with multiple pathways, including proteoglycans in cancer and chemical carcinogenesis−DNA adducts. Interestingly, the IL‐17 signaling pathway, which has been implicated in tumor‐related inflammation and immune cell recruitment, was also found to be enriched (Figure 6E). These results suggest that SRM expression is associated with tumor development and may be involved in the IL‐17 signaling pathway.

### 3.7. SRM and IL‐17 Signaling Pathway Verification

**Figure 7:**
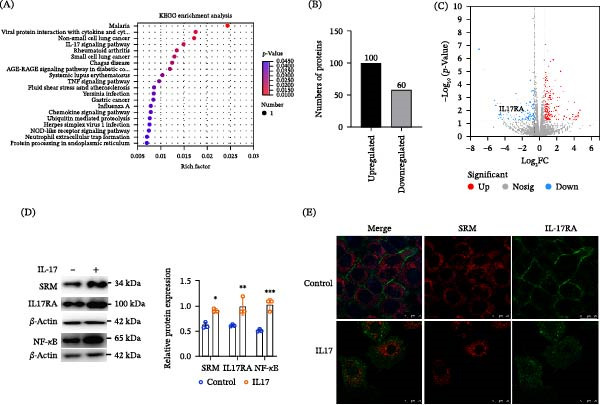
SRM and IL‐17 signaling pathway variation. (A) KEGG enrichment plot from the transcriptomic analysis of the siNC and SRM‐knockdown groups. (B) A total of 160 DEGs were identified, with 100 upregulated and 60 downregulated. (C) The volcano plot showing IL‐17RA as a significantly downregulated gene. (D) Western blot analysis showing decreased IL‐17RA and NF‐κB levels were decreased in the HepG2 cells exposed to 50 ng/mL IL‐17. Comparison between siNC and SRM‐knockdown groups. Data were presented as mean ± SD from *n* = 3 replicates;  ^∗^ *p* < 0.05,  ^∗∗^ *p* < 0.01,  ^∗∗∗^ *p*  < 0.001. (E) Immunofluorescence analysis of SRM and IL‐17RA in HepG2 cells exposed to 0–50 ng/mL IL‐17 for 24 h. SRM (red), IL‐17RA (green), and nucleus (blue); *n* = 3, scale bar: 25 μm.

To explore the downstream signaling of SRM, we performed transcriptomic sequencing upon SRM knockdown. Compared with the control group, SRM knockdown resulted in 100 upregulated and 60 downregulated genes (Figure 7B). KEGG pathway analysis of these differentially expressed genes revealed significant enrichment of the IL‐17 signaling pathway (Figure 7A), and among the downregulated genes, IL‐17RA was identified (Figure 7C). This suggests that SRM may positively regulate IL‐17RA expression. Given that IL‐17 signaling can also regulate its own receptor and downstream effectors, we next asked whether IL‐17 stimulation could reciprocally modulate the SRM expression. Indeed, treatment with IL‐17 upregulated protein levels of SRM, IL‐17RA, and the classic downstream effector NF‐κB (Figure 7D,E). Our findings suggest that SRM both responds and contributes to IL‐17‐mediated inflammation in HCC.

### 3.8. Activation of SRM Regulates IL‐17A–Induced Act1–TRAF6 Complex Assembly and NF‐κB Signaling Pathway Activation in HCC Cells

**Figure 8:**
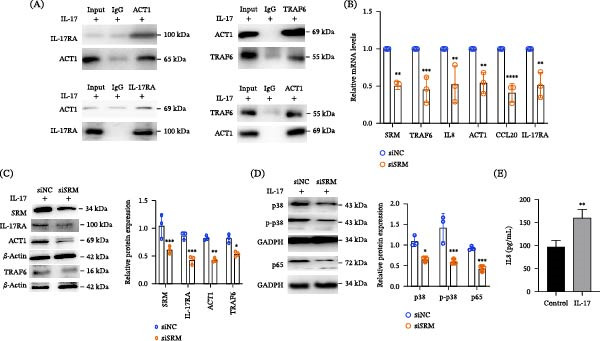
Involvement of SRM in IL‐17 A‐induced Act1–TRAF6 complex formation and NF‐κB signaling pathway activation in HCC cells. (A) Co‐IP assays of IL‐17RA interaction with ACT1 and TRAF6. (B) RT‐qPCR analysis of key members of the IL‐17 signaling pathway. The data were presented as the means ± SD; *n* = 3.  ^∗^ *p* < 0.05,  ^∗∗^ *p* < 0.01,  ^∗∗∗^p < 0.001,  ^∗∗∗∗^p < 0.0001 as compared with the cells transfected with siNC. (C) Western blot analysis showing decreased protein levels of IL‐17RA, Act1, and TRAF6 in HepG2 cells exposed to 50 ng/mL IL‐17. Comparison between siNC and SRM‐knockdown groups is shown. (D) Western blot analysis showing decreased protein levels of p38, phospho‐p38 (p‐p38), and p65 in HepG2 cells exposed to 50 ng/mL IL‐17. Comparison between siNC and SRM‐knockdown groups is shown. (E) IL‐8 levels in the supernatant of HepG2 cells following IL‐17 treatment, measured by ELISA assay. Data are presented as mean ± SD (*n* = 3).  ^∗^ *p*  < 0.05,  ^∗∗^ *p*  < 0.01,  ^∗∗∗^ *p*  < 0.001 vs. siNC group.

Given that the proximal signaling complex of IL‐17R (comprising IL‐17R, Act1, and TRAF6) is essential for IL‐17‐driven NF‐κB activation, we further examined whether SRM regulates the assembly of this complex. Co‐IP confirmed the association among IL‐17R, Act1, and TRAF6 in HCC cells, which is consistent with the formation of the proximal IL‐17R signaling complex (Figure 8A). IL‐17 robustly induced the expression of pro‐inflammatory cytokines and chemokines, including IL‐8 and CCL20, in HCC cells. SRM knockdown markedly attenuated IL‐17–driven upregulation of these genes (Figure 8B,C). Furthermore, SRM depletion significantly inhibited IL‐17–induced NF‐κB activation (Figure 8D). In vitro studies revealed IL‐17 induced IL‐8 production in HCC cells (Figure 8E). Collectively, these findings suggest that SRM may play a role in IL‐17–driven inflammatory responses in HCC cells, potentially via the NF‐κB pathway, which could contribute to the production of pro‐inflammatory cytokines such as IL‐8 and CCL20. These observations are limited to in vitro settings and require in vivo validation.

## 4. Discussion

Immunotherapy has undoubtedly revolutionized cancer treatment, enabling a significant proportion of patients with various types of cancer to achieve durable clinical responses [28]. However, the development of current immunotherapy approaches is limited by the lack of reliable biomarkers capable of accurately predicting patterns of immune infiltration, patient prognosis, and potential treatment responses. Recent studies have revealed an association between SRM expression and immune infiltration in cyclin E1‐driven ovarian cancer [10], but the exact role of SRM in the pathogenesis of HCC remains to be fully elucidated. In this comprehensive study, we identified SRM as a candidate gene significantly associated with immune cell infiltration in HCC through bioinformatics analysis. Furthermore, our in vitro experimental validation suggested that SRM may regulate inflammatory responses in HepG2 cells, thus providing insights into its potential role in HCC progression.

Our preliminary findings indicated that the SRM mRNA expression was significantly upregulated in HCC tissues compared to normal liver tissues. A comprehensive analysis of clinicopathological characteristics revealed a significant positive correlation between SRM expression levels and key parameters of HCC progression, including tumor stage, histological grade, and depth of invasion. Notably, elevated SRM expression was associated with a poor clinical prognosis. Univariate Cox regression analysis indicated that the SRM expression was associated with OS, and multivariate analysis confirmed SRM as an independent prognostic factor. These results suggest that SRM may serve as a potential prognostic marker for HCC, warranting further validation in independent cohorts.

The TME is a complex ecosystem composed of various cell populations, including immune infiltrates (such as mononuclear phagocytes, T cells, dendritic cells, B cells, and mast cells), cancer‐associated fibroblasts (CAFs), and vascular endothelial cells, which play a critical role in the development, progression, and metastasis of various malignant tumors [29, 30]. Our study indicated that SRM was positively correlated with immune infiltration. Notably, we observed that the SRM expression was negatively correlated with the levels of infiltration of native B cells, monocytes, and resting CD4^+^ memory T cells. However, while CIBERSORT revealed a negative correlation between SRM and naive B cells (*R* = −0.45, *p* < 0.001), TIMER analysis of total B cells showed a weak positive correlation (*R* = 0.183, *p* < 0.001), suggesting SRM may differentially regulate B cell subsets—a possibility that warrants further investigation using flow cytometry or single‐cell RNA sequencing.

SRM expression was significantly associated with immune infiltration, ICP TMB, and MSI in HCC, suggesting a potential link between SRM and ICP regulation. However, further functional studies are needed to determine whether SRM plays a causal role in ICP gene activation. Additionally, we observed that SRM expression was correlated with TMB and MSI, two established predictors of immunotherapy response, indicating that SRM warrants further evaluation as a potential biomarker. Of note, when SRM inhibitors (MCHA compounds) are used in bladder cancer models in combination with targeted therapy, they have demonstrated encouraging efficacy [31]. This precedent suggests that SRM inhibition merits further investigation in HCC, either as monotherapy or in combination with ICP inhibitors, although such applications remain speculative without direct experimental evidence in HCC models. Future studies should address several limitations: while our correlation analysis has established a preliminary association between SRM and ICP regulation, comprehensive mechanistic studies using well‐designed in vivo experiments are necessary to elucidate the underlying molecular pathways and validate the therapeutic potential of SRM.

To elucidate the functional role of SRM, we constructed a comprehensive protein–protein interaction network. The results indicated that multiple oncogenes and immune‐related genes may be potential interacting partners of SRM. Notably, the predictions revealed associations between SRM and ATAD3A, ATAD3B, UBE2J2, ENO1, and MRTO4, all of which have previously been implicated in the regulation of immune responses and tumorigenesis. To explore the potential role of SRM in immune infiltration, we performed functional analyses using GO and KEGG pathway enrichment approaches. The results indicate that SRM‐associated genes are enriched in the IL‐17 signaling pathway, a key regulator in immune‐oncology [32]. Previous studies have demonstrated that the IL‐17 signaling pathway recruits myeloid suppressor cells, which promote angiogenesis while suppressing antitumor immune responses. Evidence from multiple studies indicates that elevated levels of IL‐17A‐producing immune cells—including innate lymphoid cells [33], CD4^+^ T cells [34], and IL17A^+^ neutrophils [35] are associated with poor clinical outcomes. To explore this further, we conducted in vitro experiments to examine the role of the SRM in IL‐17 signaling. In HepG2 cells, we observed an upregulation of both SRM expression and IL‐17 levels. Notably, SRM knockdown resulted in attenuated activation of IL‐17‐associated signaling, suggesting that SRM may play a regulatory role in this pathway. However, the specific mechanism remains unclear, and these findings are limited to a single cell line. Our in vitro findings suggest that SRM may be involved in the assembly of the IL‐17R‐Act1‐TRAF6 signaling complex in HCC cells. IL‐17 induces IL‐8 production, which has been associated with immunosuppressive cell recruitment and immunotherapy resistance in HCC [36]. Collectively, these findings suggest that targeting the SRM‐IL‐17 axis represents a candidate pathway for further investigation as a potential therapeutic target in HCC, although this hypothesis requires validation in in vivo models and clinical samples.

Several limitations of this study should be acknowledged. First, the bioinformatics analyses were based on publicly available data, and the correlation between SRM expression and immune infiltration requires validation in independent cohorts. Second, the in vitro experiments were limited to a few cell lines, such as HepG2, and the results may not be generalizable to other HCC cells or the in vivo context. Third, the current data only suggest an association between SRM and the IL‐17 pathway, lacking direct causal evidence from in vivo knockdown or knockout models. Fourth, the therapeutic potential of targeting SRM remains speculative and requires functional validation using orthotopic HCC models and patient‐derived xenograft models.

## 5. Conclusion

This study identifies correlations between SRM expression and immune‐related features in HCC and provides in vitro evidence linking SRM to the IL‐17 signaling pathway. However, these findings are preliminary and lack in vivo validation. Therefore, the proposed roles of SRM in immune modulation and inflammation remain speculative.

## Author Contributions

Xue‐mei Wang and Ya‐nan Li designed this study. Bo‐wen Wu performed the bioinformatics analysis and performed experiments. Feng‐hong Wang performed the bioinformatics analysis and analyzed the data. Lei Zhang and Ting Li made important contributions to data acquisition and integration. Jian‐qiang Zhang and Sheng‐bo Yang drafted the manuscript. Xue‐mei Wang and Ya‐nan Li reviewed and revised the manuscript.

## Funding

This work is supported by the Research Initiation Grant for Talent Introduction to Inner Mongolia Medical University (Grant DC2400000598).

## Disclosure

All authors contributed to this paper and approved the submitted version.

## Ethics Statement

For the bioinformatics analysis, publicly available and identified data from the GEO database and TCGA database were used, the use of which was approved.

## Consent

Informed consent was not required for this study, as the research used publicly available genomic data.

## Conflicts of Interest

The authors declare no conflicts of interest.

## Supporting Information

Additional supporting information can be found online in the Supporting Information section.

## Supporting information


**Supporting Information 1** Table S1: The prognostic accuracy and risk score of SRM.

**Supporting Information 2** Table S2: Coexpression analysis between enriched genes interacting with SRM.

**Supporting Information 3** Table S3: The immune score and stem cell score of SRM.

**Supporting Information 4** Table S4: The immuno‐infiltration of SRM.

**Supporting Information 5** Table S5: The correlation analysis of SRM and immune cells.

**Supporting Information 6** Table S6: Genes associated with immune checkpoints through the Wilcoxon test.

**Supporting Information 7** Table S7: Spearman’s correlation analyses of TMB.

**Supporting Information 8** Table S8: Spearman’s correlation analyses of MSI.

**Supporting Information 9** Table S9: siRNA sequences are detailed.

**Supporting Information 10** Table S10: All primers in real‐time qPCR were listed.

**Supporting Information 11** Figure S1: Positive coexpression proteins with SRM. SRM showed a top positive correlation with ABCF2, ADPRS, AGTRAP, ATAD3A, ATAD3B, ATP6V0B, ATP6V1F, CCDC124, CD320, EIF3B, EIF3I, ENO1, FAAP20, G6PD, HSPBP1, INTS11, JPT1, LSM10, LYPLA2, MIIP, MRPL20, MRTO4, NOC2L, NUDC, NUTF2, PGD, PPP1R14B, PTDSS2, PUSL1, RCC1, RRP9, SLC52A2, SLC66A1, SNRPA, SSU72, SZRD1, THAP3, TOMM40, UBE2J2, UBE2M, UBE2S, YARS1, ZBTB8OS, ZBTB17, and ZCCHC17.

## Data Availability

All the data are available from the corresponding author upon reasonable request.
