# Supplementary material for: SRM Represents a Novel Prognosis Biomarker and Correlates With Inflammation and Immune Infiltration in Hepatocellular Carcinoma
Source: Mediators Inflamm. 2026 Jul 13;2026:6025289. doi: 10.1155/mi/6025289 (PMC13359114; doi:10.1155/mi/6025289)
Supplement: Supplementary file 11 — Supporting Information 11 Figure S1: Positive coexpression proteins with SRM. SRM showed a top positive correlation with ABCF2, ADPRS, AGTRAP, ATAD3A, ATAD3B, ATP6V0B, ATP6V1F, CCDC124, CD320, EIF3B, EIF3I, ENO1, FAAP20, G6PD, HSPBP1, INTS11, JPT1, LSM10, LYPLA2, MIIP, MRPL20, MRTO4, NOC2L, NUDC, NUTF2, PGD, PPP1R14B, PTDSS2, PUSL1, RCC1, RRP9, SLC52A2, SLC66A1, SNRPA, SSU72, SZRD1, THAP3, TOMM40, UBE2J2, UBE2M, UBE2S, YARS1, ZBTB8OS, ZBTB17, and ZCCHC17. [file MI-2026-6025289-s006.docx]

Supplementary Materials for

**SRM represents a novel prognosis biomarker and correlates with inflammation and immune infiltration in hepatocellular carcinoma (HCC)**

Bo-wen Wu^1#^, Feng-hong Wang^1#^, Lei Zhang^4^,Ting Li^2^, Jian-qiang Zhang^2^,Sheng-boYang^2^, Xue-mei Wang^2*^, Ya-nan Li^3*^

Correspondence to: Ya-Nan Li: 20230046@immu.edu.cn

**These materials include:**

Figures S1

Tables S1 to S10

**Supplementary Figure S1**


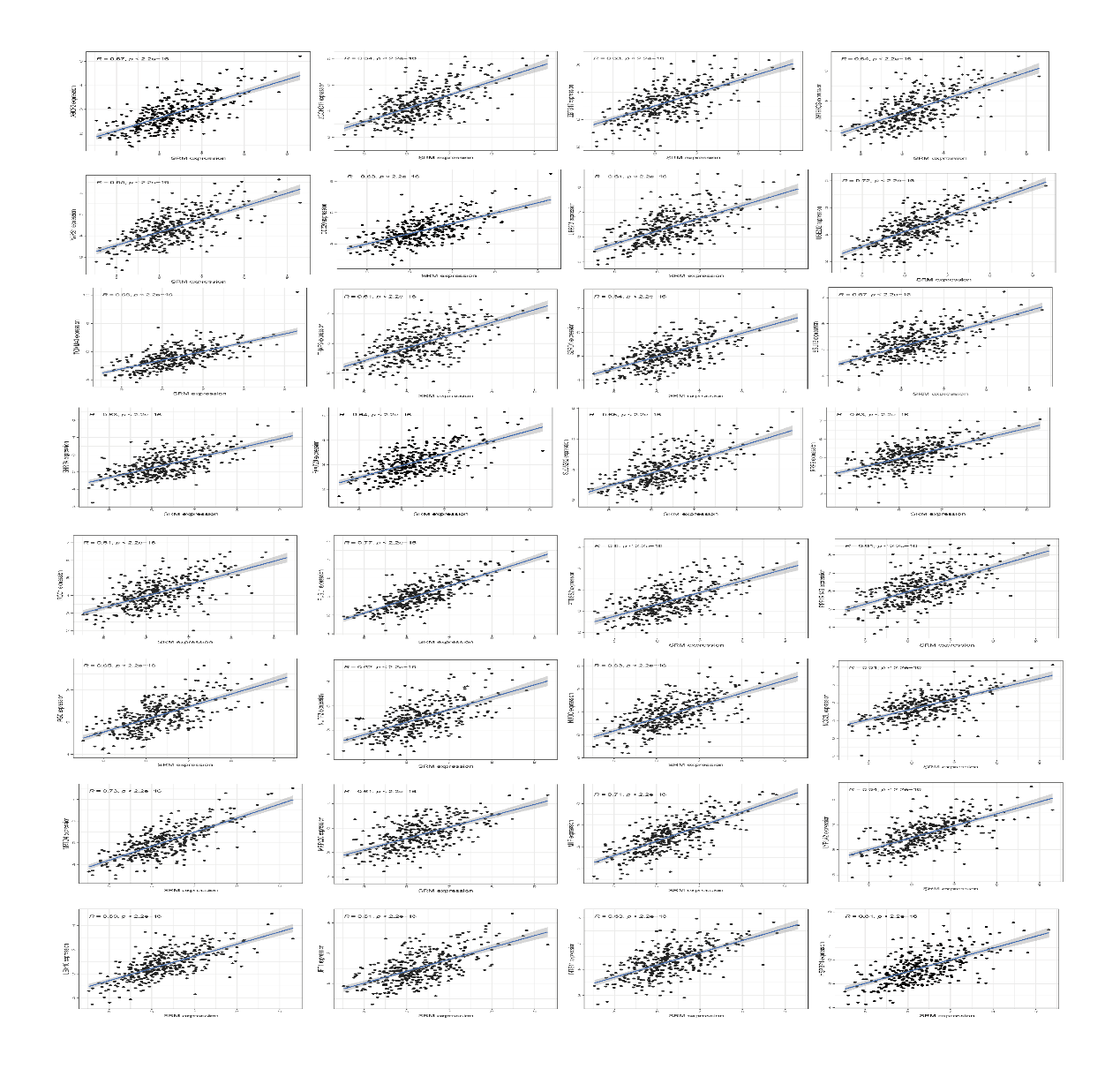


**Figure S1. Positive co-expression proteins with SRM**

**S**RM showed a top positive correlation with ABCF2, ADPRS, AGTRAP, ATAD3A,

ATAD3B, ATP6V0B, ATP6V1F, CCDC124, CD320, EIF3B, EIF3I, ENO1, FAAP20, G6PD,HSPBP1,INTS11,JPT1,LSM10,LYPLA2,MIIP,MRPL20,MRTO4,NOC2L,NUDC,NUTF2,PGD,PPP1R14B,PTDSS2,PUSL1,RCC1,RRP9,SLC52A2,SLC66A1,SNRPA,SSU72,SZRD1,THAP3,TOMM40,UBE2J2,UBE2M,UBE2S,YARS1,ZBTB8OS,ZBTB17,ZCCHC17.

**Table S1** Using the “survival and survminer” package obtained the Kaplan-Meier curves and using an R package “timeROC” analysis performed to compare the prognostic accuracy and risk score of SRM.

**Table S2** Co-expression analysis was used in the “ggplot, ggpur and ggExtra” packages, and co-expression circle diagram R package “corrplot and circlize” analysis was performed to enrich genes interacting with SRM.

**Table S3** The “estimate” package to estimate SRM of the immune score and stem cell score.

**Table S4** The immuno-infiltration results file via the install “preprocess Core” package.

**Table S5** Correlation analysis of SRM and immune cells was analyzed by co-expression analysis documents.

**Table S6** Genes associated with immune checkpoints through the Wilcoxon test.

**Table S7** Spearman’s correlation analyses of TMB.

**Table S8** Spearman’s correlation analyses of MSI

**Table S9** siRNA sequences are detailed.

**Table S10** All primers in real-time qPCR were listed.
